# Surgery for skeletal metastases in lung cancer: Complications and survival in 98 patients

**DOI:** 10.3109/17453674.2011.552779

**Published:** 2011-02-10

**Authors:** Rudiger J Weiss, Rikard Wedin

**Affiliations:** Department of Molecular Medicine and Surgery, Section of Orthopaedics and Sports Medicine, Karolinska University Hospital, Karolinska Institutet, Stockholm, Sweden

## Abstract

**Background and purpose:**

Most lung cancer patients with skeletal metastases have a short survival and it is difficult to identify those patients who will benefit from palliative surgery. We report complication and survival rates in a consecutive series of lung cancer patients who were operated for symptomatic skeletal metastases.

**Methods:**

This study was based on data recorded in the Karolinska Skeletal Metastasis Register. The study period was 1987–2006. We identified 98 lung cancer patients (52 females). The median age at surgery was 62 (34–88) years. 78 lesions were located in the femur or spine.

**Results:**

The median survival time after surgery was 3 (0–127) months. The cumulative 12-month survival after surgery was 13% (95% CI: 6–20). There was a difference between the survival after spinal surgery (2 months) and after extremity surgery (4 months) (p = 0.03). Complete pathological fracture in non-spinal metastases (50 patients) was an independent negative predictor of survival (hazard ratio (HR) = 1.8, 95% CI: 1–3). 16 of 31 patients with spinal metastases experienced a considerable improvement in their neurological function after surgery. The overall complication rate was 20%, including a reoperation rate of 15%.

**Interpretation:**

Bone metastases and their subsequent surgical treatment in lung cancer patients are associated with high morbidity and mortality. Our findings will help to set appropriate expectations for these patients, their families, and surgeons.

Lung cancer has become one of the most common cancers worldwide and is the predominant cause of death among cancer patients. The American Cancer Society estimated that almost 160,000 patients would die from lung and bronchus cancer in the USA in 2009 (Jemal et al. 2009). Some authors have stated that lung cancer is one of the most important challenges in oncology at the present time (Boyle and Dresler 2005).

Historically, about one third of all lung cancer patients are found to have bone metastases during the course of the disease. Symptoms and events of skeletal origin such as pain, pathological fractures, spinal cord compression with paraparesis, and hypercalcemia are common complications. The decline in quality of life and eventual death of these patients can be explained to some extent by skeletal complications and their treatment (Coleman 1997).

Lung cancer patients with skeletal events have a short expected survival; however, some case reports have involved patients who survived several years after pathological fractures (Agarwala and Hanna 2005, Hirano et al. 2005). A major problem in selecting patients for surgery is to avoid operating on those who are likely to die very soon after surgery. Although several features help to identify patients with long survival (Bauer and Wedin 1995, Tomita et al. 2001), it is still difficult to identify those who will die early.

We analyzed a consecutive series of lung cancer patients who were operated on for skeletal metastases at our department, to determine the complications and reoperation rates after surgery and to identify risk factors for early death.

## Patients and methods

This study was based on data recorded in the Karolinska Skeletal Metastasis Register (Wedin and Bauer 2005). The register is a quality-control database that prospectively collects individual-based information for cancer patients admitted to Karolinska University Hospital in Stockholm. All data are collected based on the national registration number (a 10-digit number), which is unique for each Swedish resident. The criterion for inclusion in the database is surgical treatment for complete or impending fractures due to skeletal metastasis.

The register gathers data on patient identity, age, sex, primary tumor, location of metastases, type of pathological fracture, and surgical treatment such as method of fixation, type of implant, and postoperative complications. It includes staging information for patients diagnosed with lung cancer (Mountain 1997). Neurological function in patients with spinal metastases is assessed by the Frankel classification of motor and sensory compromise (Frankel et al. 1969, Davis et al. 1993). The neurological function is assessed preoperatively and within 2 weeks postoperatively.

For the present study, we included all lung cancer patients who had surgery due to pathological fractures. None of the patients were excluded for any reason. In patients who had surgery for more than 1 metastasis, all sites were included in the analysis. However, only the first surgical procedure was accounted for in the survival analysis. The study period was 1987–2006.

We identified 98 individual lung cancer patients (52 females) treated surgically for skeletal metastatic lesions. The median age at surgery was 62 (34–88) years. Most patients in this cohort (n = 88) were current or former smokers at diagnosis of the disease. Adenocarcinoma was found in most cases (n = 58) followed by squamous cell carcinoma (n = 14) (Table 1). The lung cancer patients were grouped in the following stages: 11 IA + IB, 1 IIA + IIB, 5 IIIA, 73 IIIB + IV, and such data were missing for 8 patients.

**Table 1.** 98 lung cancer patients with skeletal metastases

	Total	Males	Females
Number of patients	98	46	52
Median age at diagnosis (years)	62	60	63
range	34–88	43–88	34–84
Smoking status at diagnosis			
Former smoker	47	21	26
Current smoker	41	20	21
Never smoker	10	5	5
Histopathology			
Non-small cell cancer			
Adenocarcinoma	58	25	33
Squamous cell carcinoma	14	8	6
Low differentiated non-small cell cancer	8	4	4
Large cell carcinoma	7	3	4
Small cell cancer	6	3	3
Other cancers	5	3	2
Skeletal metastasis			
Femur	46	18	28
Vertebra	31	24	7
Humerus	16	2	14
Other	8	2	6
Type of surgical procedure			
Internal fixation	34	10	24
Endoprosthesis	32	11	21
Spinal decompression + instrumentation	17	12	5
Spinal decompression	15	12	3
Other	3	1	2
Preoperative treatment (primary tumor)			
Chemotherapy	52	24	28
Radiotherapy	27	10	17
Lobectomy	12	5	7
Postoperative treatment (bone metastasis)			
Radiotherapy	59	28	31

### Statistics

Median values and ranges were used as descriptive statistics. Kaplan-Meier analysis was used to construct the cumulative survival with 95% confidence intervals (CIs). The time between diagnosis of lung cancer and death and time between surgical procedure and death were included in the survival analysis. The log-rank test was used to compare survival after extremity surgery with spine surgery.

The Cox multiple-regression model was used to study risk factors for death related to the patient and to the surgical procedure. The results were expressed as hazard ratios (HRs) with corresponding 95% CI. If a HR was > 1, the patients at risk were dying at a faster rate than the patients in the reference group. The assumption of proportional hazards was investigated by hazard function plots and log-log plots for all covariates. No signs of insufficient proportionality were detected in the hazard functions and the log-log plots ran parallel for all covariates. The factors studied in the univariate Cox model were as follows: age, sex, smoking status, type of lung cancer, staging, location of skeletal metastasis, type of pathological fracture, preoperative hemoglobin levels, time period of surgery (1987–1996 vs. 1997–2006) and perioperative chemo- and radiotherapy (Table 1). Any variable whose univariate test had a p-value of < 0.25 was considered as a candidate for the multivariate model along with all variables of known biological importance. Wilcoxon's signed ranks test was used to compare preoperative and postoperative neurological function in patients with spine metastases. The level of significance was set at p ≤ 0.05. All statistical analyses were performed using the PASW statistics package version 18 (SPSS Inc., Chicago, IL).

## Results

Most metastatic bone lesions were located in the femur and vertebra. Of the 31 vertebral lesions, 22 were located in the thoracic spine and 9 in the lumbar spine. 53 of 70 non-spinal lesions had caused complete pathological fractures. Regarding treatment of the primary tumor, more than half of the patients (n = 52) had chemotherapy and 27 had radiotherapy. Postoperative radiotherapy for the operated lesions was registered in 59 patients (Table 1). None of the patients were lost to follow-up, and none were still alive at the time of this analysis.

The neurological function in patients with spine metastases improved after surgery (p = 0.001). 16 of the 31 patients gained at least 1 Frankel grade, 14 maintained their neurological function, and 1 patient deteriorated (Table 2). The main indication for surgery in these patients was spinal cord compression (n = 29) or painful instability (n = 2).

**Table 2.** Pre- and postoperative neurological function in 31 patients with spine metastasis graded according to Frankel

	Preop.	Follow-up
A. Complete paraplegia	2	1
B. No motor function	6	2
C. Motor function useless	14	7
D. Slight motor deficit	6	16
E. No motor deficit	3	5

### Survival analysis

The median survival time after surgery of skeletal metastatic lesions was 3 (0–127) months. Males had a median survival time of 2 (0–59) and females of 4 (0–127) months (Table 3). The cumulative 6-, 12-, and 18-month survival after surgery was 24% (CI: 15–33), 13% (CI: 6–20), and 6% (1–11) respectively (Figure).

**Table 3.** Median survival of lung cancer patients with skeletal metastases. Time in months (range)

	A	B
All	10 (0–168)	3 (0–127)
Males	8 (1–60)	2 (0–59)
Females	10 (0–168)	4 (0–127)
Histopathology		
Non-small cell cancer	10 (0–168)	4 (0–127)
Adenocarcinoma	11 (1–168)	4 (0–127)
Squamous cell carcinoma	9 (0–43)	2 (0–18)
Low differentiated non-small cell cancer	8 (1–12)	2 (0–8)
Large cell carcinoma	11 (6–60)	7 (0–59)
Small cell cancer	8 (1–23)	2 (0–3)
Other cancers	5 (1–24)	1 (1–15)
Spinal surgery		
All	7 (2–29)	2 (0–15)
Males	7 (2–29)	2 (0–15)
Females	8 (3–20)	1 (1–7)
Extremity surgery		
All	10 (0–168)	4 (0–127)
Males	10 (1–60)	3 (0–59)
Females	11 (0–168)	4 (0–127)
Pathological fracture		
Complete	8 (0–80)	2 (0–61)
Impending	12 (2–168)	4 (0–127)

A: time between diagnosis of lung cancer and death; B: time between surgical procedure and death.

**Figure:**
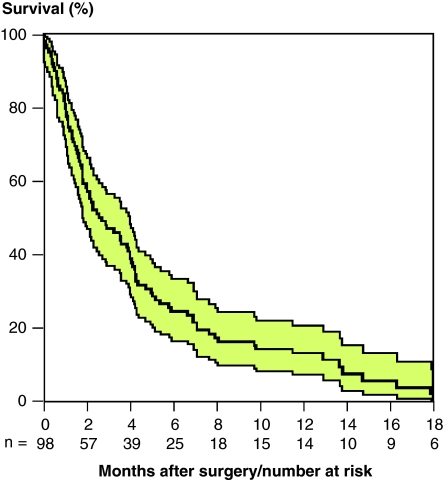
Cumulative survival of lung cancer patients after surgery for skeletal metastases, with 95% confidence intervals.

There was a difference between the median survival time after spinal surgery (2 (0–15) months) and after extremity surgery (4 (0–127) months) (p = 0.03) (Table 3).

The univariate Cox regression analysis revealed an increased risk of death after surgery for patients with complete pathological fracture, metastatic lesions in the vertebra, preoperative hemoglobin levels less than 10 g/dL, small cell cancer, and the absence of postoperative radiotherapy. The multivariate Cox analysis with the above 5 variables and age and sex showed that complete pathological fracture was a significant predictor of survival (Table 4). All the other factors studied—such as age, sex, smoking status, staging, time period of surgery, and chemotherapy—were not associated with an increased risk of death.

**Table 4.** Risk of death after surgery for skeletal metastases in lung cancer patients (Cox regression analysis)

Factor	HR	95% CI	p-value
Complete pathological fracture **^a^**	1.7 **^f^**	1.1–2.6	0.02
	1.8 **^g^**	1.0–3.0	0.05
Vertebral skeletal metastasis **^b^**	1.7 **^f^**	1.0–2.7	0.03
	1.7 **^g^**	0.9–3.4	0.1
Hemoglobin level < 10 g/dL **^c^**	1.9 **^f^**	1.1–3.4	0.03
	1.8 **^g^**	0.9–3.7	0.09
Small cell cancer **^d^**	2.6 **^f^**	1.2–6.0	0.02
	1.9 **^g^**	0.8–4.8	0.2
Postoperative radiotherapy (no) **^e^**	1.7 **^f^**	1.1–2.6	0.03
	1.6 **^g^**	0.9–2.9	0.1

Factor versus **^a^** impending pathological fracture,

**^b^** femur,

**^c^** ≥ 10 g/dL,

**^d^** adenocarcinoma,

**^e^** yes;

HR = hazard ratio, **^f^** unadjusted,

**^g^** adjusted (HRs are adjusted for type of pathological fracture, location of skeletal metastasis, preoperative hemoglobin levels, type of lung cancer, postoperative radiotherapy, age, and sex).

### Reoperations and complications

The overall complication rate including reoperations was 20/98. 15 failed reconstructions in 14 patients led to a new operation. The reasons for these reoperations were poor initial fixation, local tumor progression, periprosthetic fracture, deep infection, paraplegia, material failure, nonunion, and technical error. The median time to failure was 1 (0–13) months (Table 5).

**Table 5.** Reoperations after surgery for skeletal metastases in lung cancer patients

Sex, age, histology	Localization	Primary operation	Time (months) to and reason for failure	Treatment
F, 57, **^b^**	Humerus, proximal	Intramedullary nail	5, local tumor progression	Hemiarthroplasty
F, 63, **^c^**	Humerus, distal	Plate	3, material failure	Plate
F, 62, **^a^**	Acetabulum	Acetabular reinforcement ring + total joint arthroplasty	1, deep infection	Debridement
F, 62, **^a^**	Femur, proximal	Total joint arthroplasty	9, periprosthetic fracture	Modular tumor prosthesis
F, 74, **^a^**	Femur, proximal	Hemiarthroplasty	3, periprosthetic fracture	Modular tumor prosthesis
F, 66, **^a^**	Femur, trochanteric	Hemiarthroplasty	1, poor initial fixation	Total joint arthroplasty
M, 45, **^a^**	Femur, trochanteric	Sliding hip screw	1, poor initial fixation	Total joint arthroplasty
F, 73, **^b^**	Femur, trochanteric	Sliding hip screw	1, poor initial fixation	Exchange of screw
			4, poor bone stock	Total joint arthroplasty
F, 62, **^a^**	Femur, subtrochanteric	Hemiarthroplasty	0, technical error	Hemiarthroplasty
F, 53, **^a^**	Femur, distal	Plate	13, non-union + local tumor progression	Plate
M, 56, **^a^**	Vertebra, thoracic	Decompression	0, paraplegia (hematoma)	Removal of hematoma
M, 55, **^d^**	Vertebra, thoracic	Decompression + instrumentation	8, local tumor progression	Decompression
F, 48, **^a^**	Vertebra, thoracic	Decompression + instrumentation	0, poor initial fixation	Reinstrumentation
M, 72, **^b^**	Vertebra, thoracic	Decompression	1, deep infection	Debridement

**^a^** adenocarcinoma.

**^b^** squamous cell carcinoma.

**^c^** large cell carcinoma.

**^d^** other.

Complications that were treated non-surgically were seen in 5 of the patients, including wound infection, gastric bleeding, poor initial fixation, and technical error (Table 6).

**Table 6.** Complications after surgery for skeletal metastasis in lung cancer patients

Sex, age, histology	Localization	Primary operation	Time (months) to and reason for failure	Treatment
F, 75, **^a^**	Femur, distal	Intramedullary nail	0, fatal gastric bleeding	Non-surgical
M, 48, **^c^**	Vertebra, thoracic	Decompression + instrumentation	0, wound infection	Non-surgical
F, 48, **^b^**	Vertebra, thoracic	Decompression + instrumentation	0, gastric bleeding	Non-surgical
M, 62, **^d^**	Vertebra, thoracic	Decompression + instrumentation	0, technical error	Non-surgical
M, 55, **^d^**	Vertebra, thoracic	Decompression	1, wound infection	Non-surgical

**^a^** adenocarcinoma.

**^b^** large cell carcinoma.

**^c^** small cell lung cancer.

**^d^** other.

## Discussion

Our main finding was the short survival of lung cancer patients after surgery for skeletal metastases. Complete pathological fracture was identified as an independent risk factor for death. Moreover, a high complication rate poses a considerable burden to the patients.

The overall median survival time after surgery was only 3 months, and just 13% of the patients were still alive 1 year postoperatively. 3 of 4 patients were already classified as having an advanced stage of lung cancer at the diagnosis of the primary tumor. The 1-year survival rate of patients operated for skeletal metastases from various primary tumor sites is limited, and ranges between 30 and 54% (Bono et al. 1991, Bauer and Wedin 1995, Durr and Refior 1998, Bohm and Huber 2002, Hansen et al. 2004, Lin et al. 2007). Differences in patient selection, type of primary tumor, and indications for surgery may explain the spread in survival time. We have previously reported on the surgical outcome of 107 patients with skeletal breast cancer metastases. The postoperative median survival in these patients was 8 months (Wedin et al. 2001). Sugiura et al. (2008) reported a median survival time of 7 months in 118 patients with bone metastasis from lung cancer; however, only one third of these patients underwent surgery. Nathan et al. (2005) found that in patients with various types of cancer metastases, lung cancer patients fared the worst with a median survival time of 4 months. The short survival time has also been reported by other authors (Bauer and Wedin 1995, Durr and Refior 1998, Katagiri et al. 2005).

We found a shorter survival after operations for skeletal metastases of the spine (2 months) as compared to the extremities (4 months). Bauer and Wedin (1995) described a median 1-year survival of 25% after spinal surgery and 31% after extremity surgery, based on all types of cancer metastases. However, this difference was not statistically significant.

The decision between a surgical and non-surgical treatment of a patient with bone metastasis is influenced by the anatomical location, the tumor type, the extent of the tumor, the general medical condition of the patient, and expected survival time. Surgery may be chosen if the patient is healthy enough and life expectancy is sufficiently long to benefit from pain relief, improved mobility, and care. If there is no expectation that the survival may be long enough for the patient to recover and truly benefit from surgical procedures, other palliative therapies may be chosen instead. However, this may restrict the patients involved to bed or chair.

New therapies have been introduced to improve the survival of patients with lung cancer (Giaccone et al. 2006, Sandler et al. 2006). The median survival of patients with advanced lung cancer has increased from 6 months to 12 months with the introduction of new treatment regimens such as chemotherapy in combination with a monoclonal antibody, which acts as an angiogenesis inhibitor (Rosen et al. 2003, Sandler et al. 2006). However, the efficacy of promising new agents in lung cancer patients concerning treatment of metastases in bone, which is one of the most common metastatic sites, is unknown (Langer and Hirsh 2010).

Some authors have stated that a life expectancy of at least 2 months is usually required for meaningful surgery of limb metastases (Harrington et al. 1976), and of 3 to 6 months for spinal lesions (Cybulski et al. 1987, Atanasiu et al. 1993, Tomita et al. 1994). We think that stabilization of long-bone fractures is almost always justified unless the patient has reached a terminal stage. However, we must raise the question of whether spinal surgery would be justified in our cohort with a median survival time of barely 2 months. Decision making regarding the management of pathological fractures is complex, balancing tumor biology, biomechanics, and functional outcome goals. We believe that surgery for vertebral metastases may be the best alternative in patients who are expected to live for at least another 2–3 months if surgery leads to a substantial difference, i.e. if the patient can avoid being bedridden or can retain the ability to ambulate independently.

More than half of our patients with spine disease improved considerably, i.e. at least 1 Frankel grade. Still, a 2-month survival will be necessary in most cases to gain real benefit from the procedure, considering postoperative pain, surgical complications, and sometimes slow neurological recovery. Today, we are more cautious with patient selection for surgery because of the short survival rate in combination with a high complication rate in this cohort.

We identified low preoperative hemoglobin levels as a negative predictor of survival. Hemoglobin has been used as a prognostic factor in metastatic prostate cancer (Matzkin et al. 1993) and in heterogeneous groups of cancer patients (Hansen et al. 2004, Nathan et al. 2005). In the multivariate analysis, we could show that complete pathological fractures had an unfavorable prognosis for survival, as described by other authors (Sugiura et al. 2008).

Almost all of the patients (14/16) with humeral metastases had complete fractures. This is probably because impending fractures in non-weight bearing bones frequently escape diagnosis due to the relative absence of load-related pain.

We found a complication rate of 20%. The total failure rate leading to reoperation of previously operated metastatic sites was 15%. We have previously reported a reoperation rate of 12% in skeletal breast cancer metastases (Wedin et al. 2001) and a failure rate of 11% in patients treated for metastatic lesions of the long bones (Wedin et al. 1999). Nathan et al. (2005) described a similar reoperation rate (11%) after surgery in patients with different types of cancer. Failures are often due to local tumor progression, stress fracture, implant failure due to nonunion, or poor fixation of osteosynthetic devices in insufficient bone stock. They appear equally often in the extremities and in the spine (Yazawa et al. 1990, Bono et al. 1991), as in our series. The high reoperation rate in our study may also be partly explained by the fact that during the 20-year time frame, many orthopedic surgeons—not all sub-specialized in orthopedic oncology—performed the operations.

Our study was limited by confounding factors. Changes in diagnostics, in selection of patients, in perioperative treatment, in indications for surgery, and in methods of fixation during the 20-year study period may have influenced the results. To address the question of whether or not to operate on lung cancer patients with bone metastases, one would want to know the survival prognosis for all patients—not only the ones who (historically) have undergone surgery—as they represent a selected group of patients. This could not be investigated, however, as our register covers only surgically-treated cancer patients.

Despite its shortcomings, the present work represents the largest follow-up study of lung cancer patients after surgery for skeletal metastases. Most studies have analyzed heterogeneous populations of patients with bone metastasis. Most analyses on indications for surgery have been based on the treatment of breast and prostate cancer, which is not necessarily applicable to lung cancer patients.

In conclusion, our findings highlight the fact that surgical treatment of bone metastases in lung cancer patients is associated with high morbidity and mortality. Thus, the selection process for the (probably) few lung cancer patients who will benefit from surgical treatment is important, especially those with spinal cord compression.
